# Impact of COVID-19 on mental health of health care workers in Spain: a mix-methods study

**DOI:** 10.1186/s12889-024-17979-z

**Published:** 2024-02-14

**Authors:** J. Ripoll, X. Chela-Alvarez, E. Briones-Vozmediano, M. A. Fiol de-Roque, R. Zamanillo-Campos, I. Ricci-Cabello, J. Llobera, C. Calafat-Villalonga, M. J. Serrano-Ripoll

**Affiliations:** 1 Primary Care Research Unit of Mallorca, Palma, 07002 Spain; 2 Grup de Recerca Atenció Primària i Promoció (GRAPP-caIB) Health Research Institute of the Balearic Islands (IdISBa), Ctra. Valldemossa, Hospital Universitari Son Espases 79, Palma, 07120 Spain; 3 Research Network On Chronicity, Primary Care and Health Promotion (RICAPPS), Palma, 07120 Spain; 4 Department of Psychology, University of the Balearic Islands, Palma, 07122 Spain; 5 Departamento de Enfermería y Fisioterapia Grupo de estudios en sociedad, salud, educación y cultura (GESEC), Universidad de Lleida.Grup de Recerca en Cures en Salut (GRECS), Institut de Recerca Biomèdica (IRB) de Lleida, Lleida, 25001 Spain

**Keywords:** Health care workers, Mental health, COVID-19, Qualitative, Mix methods, Background

## Abstract

**Background:**

Spain's lockdown measures couldn't prevent the severe impact of the COVID-19 first wave, leading to high infections, deaths, and strain on healthcare workers (HCWs). This study aimed to explore the mental health impact on HCWs in the Balearic Islands during the initial months of the pandemic, the influencing factors, and the experiences of those in a COVID-19 environment.

**Methods:**

Using a mixed-methods approach, the study encompassed quantitative and qualitative elements. Cross-sectional survey data from April to June 2020 comprised HCWs who were emailed invitations. The survey covered demographics, work, clinical and COVID-19 variables, along with psychological distress and PTSD symptoms, using validated measures. Additionally, semi-structured interviews with HCWs offered qualitative insights.

**Results:**

Three hundred thirty-six HCWs averaging 46.8 years, mainly women (79.2%), primarily nurses in primary care with over 10 years of experience. Anxiety symptoms were reported by 28.8%, 65.1% noted worsened sleep quality, and 27.7% increased psychoactive drug usage. Psychological distress affected 55.2%, while 27.9% exhibited PTSD symptoms. Gender, age, experience, COVID-19 patient contact, and workload correlated with distress, PTSD symptoms, sleep quality, and psychoactive drug usage. Interviews uncovered discomfort sources, such as fear of infection and lack of control, leading to coping strategies like information avoidance and seeking support.

**Limitations:**

Static cross-sectional design, non-probabilistic sample, and telephone interviews affecting non-verbal cues, with interviews conducted during early pandemic lockdown.

**Conclusions:**

HCWs faced significant psychological distress during the pandemic's first wave, underscoring the necessity for robust support and resources to counteract its impact on mental health.

**Supplementary Information:**

The online version contains supplementary material available at 10.1186/s12889-024-17979-z.

In January 2020, the World Health Organization [1] identified a novel coronavirus causing respiratory illness in China. The WHO declared it as global pandemic on March 11. Spanish lockdown measures were initiated on March 13, aiming to curb COVID-19 transmission [2]. The first wave heavily impacted the EU, with 517,443 cases and 48,600 deaths by April 4, 2020 [3]. In those dates Spain had 57,612 hospitalized patients, 6,532 Intensive Care Units (ICU) patients and 20,852 deaths [4]. In the Balearic Islands, despite being an area of low incidence, the large increase in the number of patients caused an emergency situation in hospitals and Primary Care, creating a climate of uncertainty for healthcare workers (HCWs). The pandemic led to overwhelming patient-professional ratios, forcing untrained professionals into patient care [5]. HCWs faced constant fear of COVID-19 exposure due to inadequate Personal Protection Equipment (PPE), resulting in substantial stress and concerns [6], and about a quarter of the COVID-19 infections during the first outbreak were among HCWs [7]. A systematic review conducted by our team shows that HCWs were at especially high risk of mental health difficulties during viral epidemic [8], showing a pooled prevalence of acute stress disorder (40%), followed by anxiety (30%), burnout (28%), depression (24%), and post-traumatic stress disorder (PTSD) (13%) [9, 10].

It has been an unprecedented situation in which HCWs had to deal with high mortality rates, overload in their workplaces, working in a high-risk environment, without being prepared, or well protected [11, 12]. In addition to this, HCWs working under COVID-19 conditions were particularly vulnerable to the risk of contracting the virus and had the added concern of potentially exposing their friends and family to a heightened risk of infection [13]. So, in April 2020, the data placed Spain as one of the countries with the highest number of infections in HCWs (21.4%), with unequal representation in all the Spanish territory (12). All these factors caused a cost in their psychological well-being, especially in those who worked on the front line and who were more exposed to the risk of COVID-19 transmission, morbidity and death [6, 14]. A meta-analysis identified 25 studies of viral epidemics that examined psychological problems in HCWs who had direct contact with affected patients and HCWs who had little, or no contact (controls) [5] shows that, compared with lower risk controls, staff in contact with affected patients had greater levels of both acute or PTSD and psychological distress. Similarly, compared to non-clinical staff, front-line staff were 1.4 times more likely to feel fear and twice as likely to suffer from anxiety and depression [15], as well as burnout and compassion fatigue [16]. Many previous studies have shown that HCWs, especially nurses, with high levels of stress were more prone to develop anxiety, frustration, depression, and other psychological disorders [17]. Long work shifts with high exposure to death and various treatment demands also added to work-related stress for staff and affected their mental health [18]. This study used a mixed method research design and involved the use of both in-depth interviews and an online survey questionnaire, which offers a more comprehensive multimethod view than a design based exclusively on questionnaires.

The aim of this study was to examine (1) the impact on mental health of HCWs in the Balearic Islands during the first months (April to June 2020) of the COVID-19 pandemic, (2) the factors influencing a greater impact and (3) the experiences of HCWs working in a COVID-19 environment.

## Methods

### Design and recruitment

The present study has an exploratory mixed-methods design, including quantitative and qualitative data [19]. We present baseline results from a cross-sectional data collection.

#### Quantitative study

##### Design

Cross-sectional study through electronic survey was undertaken from April to June 2020, during the first wave of the COVID-19 pandemic.

##### Study sample and data collection

HCWs include medical doctors, nurses, midwives, nursing assistants, admission staff and others (clinical psychologists, physiotherapists, pharmacists, health managers). An email was dispatched to all HCWs, extending an invitation to participate in the survey using their official corporate email addresses. Each email comprehensively outlined the study's particulars and provided a link to access the self-administered questionnaire. Additionally, the email explicitly communicated to prospective participants that their completion of the questionnaire would be regarded as granting consent for their involvement in the study. The setting included hospitals, primary care, emergency settings and home healthcare of the Health Service of the Balearic Islands. The Health Area of Mallorca comprises four healthcare sectors (Llevant, Ponent, Tramuntana, and Migjorn), each equipped with a hospital and several Health Zones providing primary care services and emergency settings. The Ponent sector comprises 16 Health Zones and has 16 primary care health centers. In total, it covers 327,028 insured individuals (TSI). Meanwhile, the Migjorn sector consists of 14 Health Zones and 14 primary care health centers, serving 255,536 TSI. The Llevant sector has 10 Health Zones and 10 primary care health centers, providing services to a population of 138,541 TSI. Finally, the Tramuntana sector has 6 Health Zones and 6 primary care health centers, with an assigned population of 120,533 TSI.

### Variables

#### Sociodemographic and working characteristics

Sex, age, household situation during lockdown (living alone, with a partner, with a partner and children, with children, sharing with other family, recoded as ‘living alone or living with other people’), professional category (medical doctor, nurse/midwife, nursing assistant, admission staff and others), setting (primary health care, hospital/ICU, others), years of experience (grouped as 1–5 years, 6–10 years, > 10 years).

#### Clinical characteristics

Chronic disease (yes, no), tobacco consumption increased (If you are a smoker, have you increased your daily cigarette (or related product) consumption?: yes, no), alcohol consumption increased ('Regarding alcohol, have you increased the amount of alcohol you usually consume?: yes, no), self-reported depression, anxiety and other psychological issues (yes, no, in the past/current), self-reported sleep quality (how do you sleep: worse than before, as usual, better than before), psychoactive drugs consumption (yes, no), type of psychoactive drug (antidepressant, anxiolytic, hypnotic, others), extra consumption of psychoactive drugs (yes, no), effect sought in extra psychoactive drugs (hypnotic, anxiolytic, others).

#### Variables related to COVID-19

Sick leave (yes, no), contact with patients infected (yes, no), increased workload (yes, no), COVID-19 infection (yes, no, I don’t know), having been in quarantine or in isolation (yes, no), protective measures used (insufficient/very insufficient, neutral, sufficient/very sufficient) information and training received (inadequate/very inadequate, neutral, adequate /very adequate).

#### Psychological distress

Spanish version of the General Health Questionnaire-28 (GHQ-28) [20, 21]. It is a 28-item self-administered questionnaire used to identify psychological distress in the general population, divided into four subscales (somatic symptoms, anxiety and insomnia, social dysfunction and severe depression). The items are based on the 4-point Likert scale (0—not at all, 1—no more than usual, 2—rather more than usual, and 3—much more than usual). The total score ranges between 0 and 84, where higher scores refer to higher levels of psychological distress. It correctly identified 85% of “cases” with a cutting score of 6/7 (sensitivity 76.9%, specificity 90.2%), and 83% of cases with a cutting score of 5/6 (sensitivity 84.6%, specificity 82%) [21]. We considered the cut-off score of 7 points for psychological distress.

#### Posttraumatic Stress Disorder (PTSD)

Spanish version [22] of the Davidson Trauma Scale (DTS) [23] was used to obtain a general dimensional measure of trauma intensity linked with COVID-19 pandemic. It is a 17-item self-report questionnaire of posttraumatic stress symptoms. Each item corresponds to the symptoms of PTSD (items 1–4 and 17 are related to criteria B, intrusive re-experiencing, items 5–11 are linked to criteria C, avoidance and numbness; items 12–17 are related to criteria D, hyperarousal). Participants rated both frequency and severity using 5-point (0–4), Likert-type scales. Items are rated on 5-point frequency (0 = "not at all" to 4 = "every day") and severity scales (0 = "not at all distressing" to 4 = "extremely distressing"). Total sum scores can range from 0 to 136, with higher scores indicating higher levels of trauma intensity. The DTS demonstrated good internal consistency, factorial, convergent and divergent validity both in the original and in the Spanish version.The cut-off score is 40 points (PTSD symptoms > 40 points).

The dependent variables of the study were the psychological distress, presence and intensity of symptoms of PTSD, the self-reported sleep quality and the extra consumption of psychoactive drugs.

### Data analysis

A descriptive analysis of the sociodemographic, clinical and work-related variables was carried out, reporting absolute frequencies and percentages. The relationship of descriptive variables, with psychological distress, PTSD symptoms, self-perceived sleep quality and the extra consumption of psychoactive drugs was evaluated using the Chi-square test. Three multivariate logistic regression models were performed, one for psychological distress, on for PTSD symptoms and another for self reported sleeping quality, to assess the relationship between variables. We initially selected independent variables showing a statistical significance of < 0.20 in the bivariate analysis, then a logistic regression with the backward method was performed. We carried out a likelihood ratio test to assess the goodness of fit of the competing statistical models. We used Hosmer and Lemeshow test to measure goodness of fit. Statistical analysis was performed using the SPSS version 26.0 (IBM Corp., Armonk, NY, USA), and the value of statistical significance was set at *p* < = 0.05.

### Qualitative study

#### Design

We carried out an exploratory qualitative study through in-depth semi-structured interviews.

#### Study sample and data collection

All HCWs who requested psychological help in the available free telephone support were invited to participate. Both the informed consent and the interviews were audio-recorded by telephone, due to the home lockdown.

The interviews were conducted by one psychologist with experience in qualitative methods (MJS). A topic guide (Table 1) was developed based on findings from key publications in the area [6, 24]. The guide was semi-structured, deliberately non-directive and flexible to allow freely emerge emotions, perceptions and suggestions of the interviewees [25]. The interviews lasted 30–45 min and were audio-recorded from April to May of 2020.

**Table 1:** Topic guide

1. MENTAL HEALTH: HOW DO YOU FEEL?
- Feelings, emotions (anxiety, nervousness, fears)
- At a general level: concerns, what worries you the most? Sense of perceived vulnerability
- Perception of risk at work: fear of getting sick
- Fear that someone close to them will get sick, fear that they will die (has someone close to them died?)
- If you are infected: fear of infecting others. Afraid to die
- Sleep problems, eating problems
2. WHAT FACTORS DO YOU THINK MAY BE INFLUENCING HOW YOU FEEL?
2.1 Job factors:
- training on COVID-19 issues
- own role at work
- work in high-risk environments
-social isolation, confinement, quarantine
- stress at work: changes in the way of working: procedures, excessive workload, lack of resources (saturation, collapse, increased mortality)
- perception of security, threat and risk
- Absenteeism: Have you missed your job voluntarily?
2.2 Social factors
- organizational support
- support from friends and family
- stigma, social isolation and avoidance: Have other people avoided you because of your work?
- impact on life: how has your life changed since the start of the health crisis?
3. ALTRUISTIC RISK ACCEPTANCE: HAVE YOU ACCEPTED THE RISKS OF COVID-19 BECAUSE YOU WANTED TO HELP?
4. WHAT MESSAGES AND BEHAVIORS DO YOU THINK FROM YOUR LEADERS COULD HELP?
5. WHAT OTHER TANGIBLE SOURCES OF SUPPORT DO YOU THINK ARE MOST USEFUL?
6. VISION OF THE FUTURE
- Desires to take time off from work or even change jobs
- Risk / benefit assessment of your work
- Belief of obtaining something positive when the situation ends

### Data analysis

Interview audio files were transcribed verbatim. Inductive thematic analysis [26] was undertaken, and themes were identified through a 6-step process. Two researchers (MJS, XCA) independently read and re-read the data; a code tree was generated both from the data (inductively) and from the study objectives (deductively) (Additional data 1). The codes were then applied to sentences or paragraphs. The researchers discussed the analysis of each code and grouped similar codes into categories (MJS, XCA). From this process, themes emerged and were reviewed, defined, named, and used to produce the report. To ensure internal validity, both researchers encoded and analyzed the transcriptions separately. Software NVivo11 was used to assist in the analysis.

## Results

### Findings from quantitative data

The sample consisted of 336 HCWs from the Health Service of the Balearic Islands, who voluntarily agreed to participate. The response rate could not be calculated. The sociodemographic and working characteristics are shown in Table 2. The mean age of the participants was 46.8 years old (range 21- 67), 79.2% were women, 38.5% were nurses (or midwives), 61.6% working in primary care and 82.8% with more than 10 years of work experience. Our HCWs sample is similar than reported by National Statistics Institute, especially in medical doctors and nurses [27].

**Table 2:** Sociodemographic and working characteristics of the participants

	n	%
	*N* = 336	
**Sex**		
Women	266	79.2
Men	70	20.8
**Age (years)**		
< 35	56	16.7
35–44	87	25.9
45–54	106	31.5
> = 55	87	25.9
**Live with**		
Alone	46	14.7
With other people	267	85.3
**Have children**		
yes	211	62.8
**Professional category**		
Medical doctor	93	27.8
Nurse/midwife	129	38.5
Nursing assistant	23	6.9
Admission staff	42	12.5
Others^a^	48	14.3
**Workplace**		
Primary health care (including Home Care and Emergencies)	199	61.6
Hospital/Intensive Care Unit	103	31.9
Others	21	6.5
**Years’ work experience**		
1–5	22	6.6
6–10	36	10.7
> 10	277	82.7

^a^Others includes clinical psychologists, physiotherapists, pharmacists and health managers

Concerning the COVID-19 pandemic's impact on working conditions, 64.3% of participants reported direct contact with COVID-19 infected patients, while 45.8% experienced an escalated workload. Specifically, 15% underwent quarantine or isolation due to COVID-19-compatible symptoms. Specifically, during the questionnaire response period, confirmatory COVID-19 tests were unavailable, leaving 48.2% uncertain about infection status. On the topic of protective measures, 28.2% deemed measures inadequate, and 22% found received information and training to be insufficient.

Table 3 shows the participants clinical characteristics. Up to 28.8% self-reported that they suffered from anxiety during the first wave of the pandemic and 65.1% said they slept worse than before the pandemic. Up to 27.7% answered that they needed an extra consumption of psychoactive drugs. A total of 55.2% presented psychological distress (GHQ-28 scale) and 27.9% presented worse PTSD symptoms (DTS scale).

**Table 3:** Clinical characteristics of the participants

	n	%
Depression		
In the past	69	21.6
Currently	22	6.9
Anxiety		
In the past	97	30.4
Currently	92	28.8
Other psychological problem		
In the past	20	6.3
Currently	11	3.4
How do you sleep?		
Worse than before	207	65.1
As usual	104	32.7
Better than before	7	2.2
Psychoactive drugs consumption before the pandemic (yes)	58	18.2
Type of psychoactive drugs		
Antidepressant	18	31.0
Anxiolytic	22	37.9
Hypnotic	8	13.8
Others	10	16.4
Extra psychoactive drugs consumption during the pandemic (yes)	84	27.7
Effect sought in extra psychoactive drugs		
Hypnotic	53	62.4
Anxiolytic	31	36.5
Others	1	1.2
Psychological distress (GHQ-28)		
With psychological distress	176	55.2
Without psychological distress	143	44.8
PTSD symptoms (DTS)		
With PTSD symptoms	89	27.9
Without PTSD symptoms	230	72.1

*DTS* Davison Trauma Scale, *GHQ* General Health Questionnaire, *PTSD* Post-Traumatic Stress Disorder

Table 4 shows the results of the bivariate analysis, elucidating the connections between sociodemographic variables, working characteristics, COVID-19-related factors, and psychological indicators. Notably, higher psychological distress was evident among women, individuals aged 35 to 44, and those without children. Nursing assistants, hospital/ICU staff, and with less than 5 years of experience reported more pronounced distress. Moreover, professionals who had contact with COVID-19 patients or increased their workload exhibited greater psychological distress. Similarly, women, nursing assistants, those with less than 5 years of experience, individuals with COVID-19 patient contact, and those with increased workloads displayed more PTSD symptoms. Sleep quality was notably worse among professionals aged 35 to 44, nursing assistants, and those with COVID-19 patient contact or increased workloads. However, no significant correlation was identified between the studied variables and extra consumption of psychoactive drugs.

**Table 4:** Relationship between sociodemographic, work, clinical variables and COVID-19, with GHQ, DTS, self-reported sleep quality and psychoactive drugs

	**Psychological distress**			**PTSD symptoms**			**Self-reported sleep quality**			**Extra psychoactive drugs consumption**		
	With psychological distress N (%)	Without psychological distress N (%)	***p* value**	With PTSD symptoms N (%)	Without PTSD symptoms N (%)	***p* value**	Worse than before N (%)	As usual/ better N (%)	***p* value**	**Yes** N (%)	**No** N (%)	***p* value**
**Sex**												
Women	147 (58.1)	106 (41.9)	0.03^a^	77 (30.4)	176 (69.6)	0.04^a^	169 (67.1)	83 (32.9))	0.15	71 (29.7)	168 (70.3)	0.136
Men	29 (43.9)	37 (56.1)		12 (18.2)	54 (81.8)		38 (57.6)	28 (42.4)		13 (20.3)	51 (79.7)	
**Age (years)**												
< 35	33 (62.3)	20 (37.7)	0.00^a^	19 (35.8)	34 (64.2)	0.23	35 (67.3)	17 (32.7)	0.00^a^	13 (26.5)	36 (73.5)	0.854
35–44	56 (70.0)	24 (30.0)		24 (30.0)	56 (70.0)		64 (80.0)	16 (20.0)		18 (24.7)	55 (75.3)	
45–54	54 (52.9)	48 (47.1)		29 (28.4)	73 (71.6)		60 (58.8)	42 (41.2)		28 (28.0)	72 (72.0)	
> 55	33 (39.3)	51 (60.7)		17 (20.2)	67 (79.8)		48 (57.1)	36 (42.9)		25 (30.9)	56 (69.1)	
**Live with**												
Alone	27 (55.1)	22 (44.9)	0.99	19 (38.8)	30 (61.2)	0.06	31 (63.3)	18 (36.7)	0.77	15 (17.9)	30 (13.7)	0.362
With other people	149 (55.2)	121 (44.8)		70 (25.9)	200 (74.1)		176 (65.4)	93 (34.6)		69 (82.1)	189 (86.3)	
**Have children**												
Yes	107 (51.0)	103 (49.0)	0.03^a^	49 (23.3)	161 (76.7)	0.01^a^	133 (63.6)	76 (36.4)	0.52	56 (27.7)	146 (72.3)	0.690
No	63 (64.3)	35 (35.7)		36 (36.7)	62 (63.3)		66 (67.3)	32 (32.7)		27 (30.0)	63 (70.0)	
**Professional category**												
Medical doctor	34 (38.6)	54 (61.4)	0.00^a^	16 (18.2)	72 (81.8)	0.05^a^	51 (58.0)	37 (42.0)	0.01^a^	23 (27.7)	60 (72.3)	0.765
Nurse/midwife	74 (60.2)	49 (39.8)		38 (30.9)	85 (69.1)		84 (68.9)	38 (31.1)		33 (28.2)	84 (71.8)	
Nursing assistant	20 (87.0)	3 (13.0)		10 (43.5)	13 (56.5)		22 (95.7)	1 (4.3)		8 (38.1)	13 (61.9)	
Admission staff	26 (61.9)	16 (38.1)		15 (35.7)	27 (64.3)		25 (59.5)	17 (40.5)		11 (26.8)	30 (73.2)	
Others	22 (52.1)	21 (48.8)		10 (23.3)	33 (76.7)		25 (58.1)	18 (41.9)		9 (22.0)	32 (78.0)	
**Workplace**												
PHC	95 (48.5)	101 (51.5)	0.01^a^	52 (26.5)	144 (73.5)	0.67	123 (63.1)	72 (36.9)	0.25	53 (28.6)	132 (71.4)	0.486
Hospital/ICU	70 (68.0)	33 (32.0)		32 (31.1)	71 (68.9)		73 (70.9)	30 (29.1)		28 (28.3)	71 (71.7)	
Others	11 (55.0)	9 (45.0)		5 (25.0)	15 (75.0)		11(55.0)	9 (45.0)		3 (15.8)	16 (84.2)	
**Work experience**												
< 5 years	18 (85.7)	3 (14.3)	0.00^a^	10 (47.6)	11 (52.4)	0.05^a^	16 (76.2)	5 (23.8)	0.30	7 (35.0)	13 (65.0)	0.650
6—10 years	22 (64.2)	12 (35.3)		12 (35.3)	22 (64.7)		24 (72.7)	9 (27.3)		10 (31.3)	10 (31.3)	
> 10 years	136 (51.5)	128 (48.5)		67 (25.4)	197 (74.6)		167 (63.3)	97 (36.7)		67 (26.7)	67 (26.7)	
**COVID patients contact**												
Yes	117 (60.0)	78 (40.0)	0.03^a^	64 (32.8)	131 (67.2)	0.00^a^	137 (70.6)	57 (29.4)	0.00^a^	55 (29.6)	131 (70.4)	0.412
No	50 (47.2)	56 (52.8)		19 (17.9)	87 (82.1)		58 (54.7)	48 (45.3)		25 (25.0)	75 (75.0)	
**Increased workload**												
Yes	92 (63.0)	54 (37.0)	0.01^a^	53 (36.3)	93 (63.7)	0.00^a^	109 (74.7)	37 (25.3)	0.00^a^	43 (30.7)	97 (69.3)	0.281
No	84 (48.6)	89 (51.4)		36 (20.8)	137 (79.2)		98 (57.0)	74 (43.0)		41 (25.2)	122 (74.8)	

*DTS* Davison Trauma Scale, *GHQ* General Health Questionnaire, *PHC* Primary Health Care, *PTSD* Post-Traumatic Stress Disorder

^a^Others includes clinical psychologists, physiotherapists, pharmacists and health managers

In the multivariate analysis, professionals aged 35 to 44, nursing assistants, those with fewer years of experience, and those in contact with COVID-19 patients, demonstrated heightened psychological distress. Women, HCWs in contact with COVID-19 patients, and those with increased workloads were found to be more likely to experience PTSD symptoms. Professionals aged 35-44, those in contact with COVID-19 patients and those with increased workloads, declared they slept worse than before the pandemic. Odds ratios (ORs), crude and adjusted, 95% confidence intervals (CIs), and *p*-values for each association and Hosmer and Lemeshow test are reported in Table 5.

**Table 5:** Multivariate logistic regression analysis

		**Crude OR (95% CI)**	***P* value**	**Adjusted OR (95% CI)**	***P* value**
**Multivariate logistic regression analysis of factors associated with psychological distress**					
**Sex**					
Men		1	0.041	1	0.186
Women		1.76 (1.02–3.05)		1.52 (0.81–2.87)	
**Age (years)**					
< 35		1	0.001	1	0.001
35–44		1.41 (0.68–2.94)	0.354	3.19 (1.19–8.57)	0.021
45–54		0.68 (0.34–1.34)	0.268	1.62 (0.62–4.21)	0.320
> = 55		0.39 (0.19–0.79)	0.009	0.73 (0.26–2.03)	0.550
**Professional category**					
Medical doctor		1	0.001	1	0.001
Nurse/midwife		2.39 (1.36–4.20)	0.002	2.36 (1.25–4.44)	0.008
Nursing assistant		10.58 (2.92–38.35)	0.000	10.12 (2.53–40.44)	0.001
Admission staff		0.58 (1.21–5.49)	0.014	4.64 (1.91–11.31)	0.001
*Others		1.66 (0.79–3.47)	0.175	2.24 (0.93–5.37)	0.071
**Years’ work experience**					
> 10		1	0.011	1	0.027
6–10		0.17 (0.82–3.63)	0.150	1.70 (0.61–4.77)	0.313
< 5		5.64 (1.62–19.62)	0.006	7.71 (1.72–34.51)	0.008
**Contact with COVID patients**					
No		1	0.033	1	
Yes		0.68 (1.04–2.71)	0.560	1.83 (1.04–3.23)	0.036
Hosmer and Lemeshow test, *p* = 0.888					
**Multivariate logistic regression analysis of factors associated with posttraumatic stress symptoms**					
**Sex**					
**Men**	1		0.051	1	0.038
**Women**	1.96 (0.99–3.88)			2.11 (1.04–4.26)	
**Contact with COVID patients**					
**No**	1		0.006	1	
**Yes**	2.23 (1.25–3.99)			2.25 (1.24–4.06)	0.003
**Increased workload**					
**No**	1		0.002	1	
**Yes**	0.17 (1.31–3.57)			2.12 (1.25–3.59)	0.009
Hosmer and Lemeshow test, *p* = 0.470					
**Multivariate logistic regression analysis of factors associated with self reported sleep quality**					
**Age (years)**					
< 35	1		0.009	1	0.018
35–44	1.94 (0.87–4.3)			1.91 (0.83–4.83)	
45–54	0.69 (0.34–1.39)			0.85 (0.40–1.79)	
> = 55	0.65 (0.31–1.33)			0.60 (0.28–1.29)	
**Contact with COVID patients**					
**No**	1		0.006	1	0.011
**Yes**	1.98 (1.21–3.25)			1.93 (1.16–3.22)	
**Increased workload**					
**No**	1		0.001	1	0.010
**Yes**	2.22 (1.37–3.59)			1.93 (1.17–3.20)	
Hosmer and Lemeshow test, *p* = 0.852					

**Results from qualitative data**: We conducted nine telephone in-depth interviews. Three general practitioners, one internist, one nurse, three nurse assistants, and one admission staff participated in the interviews; two interviewed were men and seven, women. Five of the interviewees worked in primary care settings, one in home care, one in an elderly residence, one in internal medicine and one in emergencies. Their mean age was 41 (range 29–51). Their experiences of working during the pandemic varied according to service type, years of experience and prevalence of COVID-19 infections.

Four main themes inductively emerged from data: 1. Sources of discomfort; 2. Strategies to cope with discomfort and sources of well-being; 3. Outcomes of discomfort; and 4. Future behavioural changes.

### Sources of discomfort

The fear of contracting or spreading COVID-19 was the most cited source of discomfort, including feelings such as anxiety, being overwhelmed, annoyed, uncertain, and sad. This fear centered around the potential of being infected or being the source of infection for loved ones or vulnerable individuals.

“At first, it really stressed me out if I got infected through my daughters, and then my parents took care of them, and ended up infecting my parents, right? That worried me a lot” (woman, GP, home care, 39y)

The perception about things being out of control and not being able to give response to all the patients, were also sources of discomfort.

“I have felt very overwhelmed, like everything was slipping away from my hands, like a nightmare (…). Then, I started hearing that some people were getting worse and dying, and the ambulances started to come. Then, the shock of seeing them with those white suits, taking people away. That was horrible” (woman, nurse assistant, geriatric residence, 42y)

All interviewees who were infected by COVID-19 or had lived close experiences (i.e. in contact with people infected) labelled the experience as very negative, tough, stressful and perceived things out of control.

“I feel like I have had post-traumatic stress, which still hasn't gone away, because I still don't feel well. It's been many days now, almost 25 days, and the stress, even before, I have tried so many things to try to relax a little, but even during the week I was admitted, I was perhaps even more stressed, more discouraged” (man, GP, 42y)

The shortage of PPE, such as masks and gloves, was another major source of discomfort. One interviewee who was responsible for managing PPE supplies expressed frustration at not being able to do their job properly. Many interviewees reported feeling unprotected and at increased risk of infection due to the lack of PPE. In fact, one participant believed that the reason they contracted COVID-19 was due to the absence of proper PPE.

“There was also the concern that we didn't have enough equipment, especially at the beginning. I was asked to prepare Personal Protective Equipment (PPE), and there wasn't enough for everyone, and I was thinking, 'How am I going to protect myself if I don't have enough?' And of course, since I was in charge (…) people would come to me. (…) It was distressing not being able to do more” (woman, nursing assistant, 29y)

Constant and drastic organizational changes at work, such as changing protocols, protective measures and procedures, and the rise of COVID-19 infected HCWs that led to a lack of personnel, caused feelings of confusion and an increase of workload as well as its complexity. The confusion and not knowing whether things were being done well, provoked perception of losing control at work.

“I think that changing the protocol so often has driven us crazy. I mean, crazy, it's like you don't know what you're thinking anymore, whether you're doing it right or doing it wrong” (woman, nursing assistant, 29y)

These organizational issues were criticized by interviewees, some of them thought that people in charge of organization should have listened to workers who worked in the front line and consider their knowledge stemmed from their front line experience to take decisions on the organization of work in health care centers.

“What is always lacking is that they listen much more to the needs of the workers and those of us who are in the field, and that they try, not only to listen but also to adopt the aid and changes to the real needs” (man, GP, 42y)

Other sources of distress less mentioned were fear of getting out of home, the drastic changes witnessed in daily life (i.e., no people neither cars in the streets), household organization when having children, not having physical contact with son/daughter and having paranoic thoughts related to pandemic.

“Of course, [I had a lot of fear] of getting infected, of the uncertainty of whether I had the virus and was coming home. I spent almost 20 days in a room in my house because I didn't know if I had the virus or not, even though I was being tested. For example, I could get tested today, but I didn't know if I had it before. So, as a precaution, I slept in a separate room at home, with separate utensils and everything, in case I had the virus. Not being able to give your daughter a kiss, 'Mom, when can I hug you?' Ugh! All of that…” (woman, nurse assistant, geriatric residence, 42y)

### Strategies to cope with discomfort and sources of well-being

Participants mentioned five ways that served them to deal with the distress explained in the previous section, including: (a) avoiding the exposure to information (TV, newspapers, social networks, talking with people); (b) taking some days-off to disconnect from work (including not talking with coworkers); (c) organizing work in order to be able to take care of children and diminish possibilities of infection of the elderly; (d) taking medication to control anxiety and, (e) sleeping in a different room in order to prevent infections.

“And now, for example, I have the TV turned off, and I don't even turn it on because… And on WhatsApp, I try not to pay attention to what people say, because if I do, I'll feel even worse” (woman, nursing assistant, 29y)

“I've had panic attacks where I couldn't cope, and I had to take alprazolam to manage them” (woman, nurse assistant, 51y)

Furthermore, some contextual factors made them feel better despite the situation they were living. Among the sources of well-being, it is noteworthy social support from family (specially important among those participants who were infected by COVID-19), friends, neighbours and even unknown people. Feeling supported at work, by coworkers, bosses or users/patients. One participant reported that daily mindfulness sessions at their hospital also contributed to their well-being. Additionally, many HCWs mentioned that being an essential service and being able to leave their homes during confinement had a positive impact on their well-being.

“It has helped me a lot, people have been very supportive, they have called me, and both my family and friends have supported me a lot” (man, GP, 42y)

### Outcomes of discomfort

Participants perceived that changes in daily life provoked by COVID-19 pandemic resulted in different negative outcomes. All participants mentioned anxiety symptoms, which sometimes were coupled with exhaustion, negative thinking, and difficulties for relaxing, eating and sleeping, even having nightmares. Other outcomes reported were the possibility of changing jobs or not wanting to go to work; taking medication to sleep and control anxiety; and PTSD of one participant being infected by COVID-19.

“At night, I had a lot of trouble sleeping, I had many nightmares (…) I saw many bodies in the morgue, suddenly a door would open and the bodies would fall out, the room numbers of the care home, I saw the elderly people (…) I slept, but had those images during my dreams” (woman, nurse assistant, geriatric residence, 42y).

### Future behavioural changes

Most participants stated that the experience helped them to appreciate the smaller things in life, living in the present moment, become better individuals or feeling the closenesses of the family; that is, daily things taken for granted, participants learned to value them more. As well, they mentioned that the pandemic made more obvious the need to prioritize health, education and environment has become apparent. One participant who had been infected mentioned the need to reduce their workload.

“And now, after what just happened to me, I have seriously considered not doing shifts if I return, because I believe that there are some moments where you have to take a break, and I think that's what I should do” (man, GP, 42y)

## Discussion

### Summary of main results

In this study we evaluated the impact on mental health of HCWs during COVID-19 first wave and explored more deeply their experiences using a mix-methods approach. The data revealed that a high percentage of HCWs reported suffering from anxiety symptoms, psychological distress, PTSD symptoms and self-reported insomnia. They have also reported a considerable increase in the need to take extra psychoactive drugs. Participants underscored some sources of discomfort (fear of being infected), explained some strategies to cope with discomfort, and narrated outcomes of discomfort (such as anxiety, exhaustion, negative thinking…), sources of well-being (I.e., social support) and outlined some future behavioral changes.

### Comparison with previous literature

This study confirms that the COVID-19 pandemic has been a traumatic event for HCWs having an impact on their psychological wellbeing. As in previous studies [6, 9, 10, 28–30], our participants showed high levels of psychological distress and symptoms of PTSD. Almost 28% of them manifested a potential diagnosis of PTSD, as in the study of Alonso et al. [31] carried out in Spain, where they found similar PTSD Figs. (22.1%), and in Martínez-Caballero et al. [28] study, where found 30.9% presented PTSD traits. A recent systematic review and meta-analysis [32] found a pooled prevalence of PTSD symptoms among HCWs higher (34%) and 14% for severe PTSD. As mentioned by interviewees, the reorganization in procedures and the work overload caused by the COVID-19 led to stress, results in line with different studies [33].

Up to 55% of participants presented psychological distress in the early phases of the pandemic, figure that is double that found in another study with Spanish professionals [28] and higher than the in the aforementioned Spanish study [31], where found figures of 45.4% for presence any mental disorder.

In our study, women and nursing assistants were the most affected in terms of psychological distress and PTSD symptoms. Also, those professionals who had fewer years of experience, those with COVID-19 patient contact, and with increased workload, as in another studies [33]. Both sexes were concerned about infecting themselves and their relatives, in line with various studies [28, 33, 34], where the figures for fear of infecting themselves (up to 94.3%) or their relatives (up to 96.8%) were very high. In a recent metanalysis [35] found up to 34 studies where the fear of contracting COVID-19 was a central key result. Moreover, our respondents were afraid not only to get infected, but also to infect their families, given that the Spanish Government did not provide alternative accommodation to HCWs at the beginning of the pandemic, not even to those directly involved in the care of patients with COVID-19. Death became present in everyday life, both in healthcare contexts and through the mass media, making the inevitability of mortality salient [36], a fact that may also increases anxiety [37]. The results of our study indicate the possibility that this fear of contagion increased in the case of having children, because if both parents worked, their children had to be cared for by third parties.

Furthermore, a notable 65% of our survey participants indicated self-reported sleep problems, a remarkably high rate, particularly when compared to the Spanish population's insomnia prevalence of 6.4% [38]. Our results indicate those aged 35 to 44, those directly involved in the care of patients with COVID-19 and with increased workload, were the most affected. In a comprehensive systematic review by Serrano-Ripoll et al. [39] encompassing 13 studies and 14,075 participants, an incidence of 38% [95%CI = 37% to 39%, I20%] of insomnia among respondents was documented. Similarly, in an investigation involving 1,379 healthcare workers in Italy [30], the reported insomnia prevalence was 21.9%. Notably, a comprehensive systematic review by Al Maqbali et al. [40] involving 18 studies evaluating sleep disturbance reported a pooled prevalence of 43% (95% CI 36–50).

It should be noted that almost 28% consumed extra psychoactive drugs, with no significant statistical relationship found among the remaining variables. Overcoming the results of a study done in Morocco and France [41], where 20% of the respondents reported that they used hypnotics and sedatives quite regularly. As qualitative results pointed out, the use of these might be explained in part by sleep disorders and anxiety. While in a clinical trial carried out at the same time, only 16.5% of the participants reported taking psychoactive drugs [42]. However, in a similar study in Spain [28], professionals slightly decreased their consumption of anxiolytics during the pandemic.

Another source of distress was work-family conciliation: those with children had to organize with whom children could stay. This fact coupled with the fear of infecting others through their children, increased their psychological distress. Despite living this stressful circumstance, HCWs also were able to identify some sources of well-being. Participants identified social support from family, friends, neighbours, and even strangers as a significant source of well-being, particularly among those who had been infected with COVID-19. Additionally, support from co-workers, bosses, and patients/users at work was identified as a source of well-being. Results in line with the results from other studies [7, 13] that recommend strategies to promote resilience and recovery from physical and mental fatigue (including briefings, supporting families, regulated recovery time during work hours, psychological first aid, and humanizing patient care).

According to the professional category, regarding psychological distress, while in our study nursing assistants and administrative staff were the most affected professionals, 60% of the nurses and those in the Galatea Foundation Report [43] had poor psychological wellbeing. Regarding the years of work, in our study it was those with fewer years of experience who presented worse psychological distress and PTSD symptoms, as in many studies, such as that of Martínez-Caballero [28], the more experience the health workers had (> 20 years), the less affected they were. In summary, results align with prior research [31], indicating high levels of distress, variable risk factors and different sources of resilience. Moreover, our HCWs sample is similar to that reported by the National Statistics Institute [27], especially in medical doctors and nurses. This similarity lends confidence to the external validity of our findings, suggesting that the experiences and challenges faced by our participants may reflect those of a broader population of healthcare professionals during the COVID-19 pandemic.

### Strengths and limitations

This research has several limitations. The first is due to the use of a descriptive cross-sectional design that only provides a static picture of the problem but can serve as a basis for longitudinal studies. The second limitation was because the sample was based on self-selection and lacked probabilistic characteristics, it was not possible to quantify invitation and response rates. In terms of limitations of the qualitative part of this study was that all participants were interviewed by telephone, because the Balearic Islands were in lockdown and the authors were unable to attend the interview in person. So, it was more difficult to obtain non-verbal cues. The dates of the interviews must be considered, it is, very early in the pandemic and in full home confinement of the entire Spanish society, which was experiencing that unprecedented situation. The last but notable limitation is the inability to calculate the response rate accurately. The recruitment approach relied on email invitations distributed to corporate distribution blind lists in healthcare settings. The absence of precise information regarding the reach and the number of HCWs who received the invitations hinders our ability to determine the response rate. This limitation introduces a degree of uncertainty regarding the representativeness of our sample and should be considered when interpreting the study findings. The main strength is the use of a mix methods approach. We used valid measurements to evaluate the impact on mental health of HCWs in the quantitative phase. An important strength of the qualitative phase is its methodological rigor. The study meets the main trustworthiness criteria: credibility, dependability, transferability and conformability [44]. The analysis categories comply with the criteria of comprehensiveness, relevancy and objectivity. Finally, most of the published studies are with doctors and nurses, while in the present study health and non-health personnel have been included.

## Conclusions

HCWs in the Balearic Islands (Spain), who served during COVID-19 pandemic encountered heightened levels of psychological distress, PTSD symptoms, anxiety and insomnia. Notably, female gender, ages between 35 and 44 years old, roles as nursing assistants, frontline work and shorter work tenure emerged as significant risk factors for diverse mental health issues. Interviewees pinpointed distress sources such as COVID-19 infection risk and disruptive workplace reorganizations fostering anxiety, overwhelm, irritation, uncertainty, and sadness. A loss of control and incomplete tasks exacerbates these sentiments, yielding outcomes like anxiety symptoms, exhaustion, negative thinking, and disrupted eating and sleeping patterns. Coping strategies encompassed shielding against excessive information exposure, taking breaks to disconnect from work, and employing psychoactive medication to manage anxiety and sleep disruptions. HCWs would gain from psychological and/or psychiatric support featuring continuous monitoring and control during and post-pandemic. This ordeal should yield a valuable lesson, underlining the necessity of addressing risk factors and devising action plans for future pandemics to alleviate suffering among our health personnel.

### Supplementary Information

**Supplementary material.**

## Data Availability

The data that support the findings of this study are available upon request (for scientific purposes) in Zenodo (https://zenodo.org/).

## References

[1] World Health Organization. Novel coronavirus (2019-nCoV). Situation Report - 1. 2020 [cited 2023 Dec 22]. Available from: https://www.who.int/docs/default-source/coronaviruse/situation-reports/20200121-sitrep-1-2019-ncov.pdf?sfvrsn=20a99c10_4.

[2] Spanish Government. Real Decreto 463/2020, de 14 de marzo, por el que se declara el estado de alarma para la gestión de la situación de crisis sanitaria ocasionada por el COVID-19. Spanish Government; 2020 Mar 14 [cited 2023 Dec 22]. Available from: https://www.boe.es/boe/dias/2020/03/14/pdfs/BOE-A-2020-3692.pdf.

[3] European Centre for Disease Prevention and Control. 2022 [cited 2023 Dec 22]. Available from: https://www.ecdc.europa.eu/en/covid-19/data.

[4] Red Nacional de Vigilancia Epidemiológica. Análisis de los casos de COVID-19 en personal sanitario notificados a la RENAVE hasta el 10 de mayo en España. Spain: Instituto de Salud Carlos III. Available from: www.isciii.es/QueHacemos/Servicios/VigilanciaSaludPublicaRENAVE/EnfermedadesTransmisibles/Paginas/Informes_Previos_Covid-19_2020.aspx.

[5] Kisely S, Warren N, McMahon L, Dalais C, Henry I, Siskind D (2020). Occurrence, prevention, and management of the psychological effects of emerging virus outbreaks on healthcare workers: rapid review and meta-analysis. BMJ.

[6] Gómez-Ochoa SA, Franco OH, Rojas LZ, Raguindin PF, Roa-Díaz ZM, Wyssmann BM (2021). COVID-19 in health-care workers: a living systematic review and meta-analysis of prevalence, risk factors, clinical characteristics, and outcomes. Am J Epidemiol.

[7] Mira JJ, Cobos-Vargas Á, Astier-Peña MP, Pérez-Pérez P, Carrillo I, Guilabert M, et al. Addressing Acute Stress among Professionals Caring for COVID-19 Patients: Lessons Learned during the First Outbreak in Spain (March-April 2020). Int J Environ Res Public Health. 2021;18(22). 10.3390/ijerph182212010. 10.3390/ijerph182212010 PMC8624221 34831767

[8] Serrano-Ripoll MJ, Meneses-Echavez JF, Ricci-Cabello I, Fraile-Navarro D, Fiol-deRoque MA, Pastor-Moreno G (2020). Impact of viral epidemic outbreaks on mental health of healthcare workers: a rapid systematic review and meta-analysis. J Affect Disord.

[9] Raoofi S, Pashazadeh Kan F, Rafiei S, Khani S, Hosseinifard H, Tajik F (2021). Anxiety during the COVID-19 pandemic in hospital staff: systematic review plus meta-analysis. BMJ Support Palliat Care.

[10] Sun P, Wang M, Song T, Wu Y, Luo J, Chen L (2021). The psychological impact of COVID-19 pandemic on health care workers: a systematic review and meta-analysis. Front Psychol.

[11] Chigwedere OC, Sadath A, Kabir Z, Arensman E. The impact of epidemics and pandemics on the mental health of healthcare workers: a systematic review. Int J Environ Res Public Health. 2021;18(13). 10.3390/ijerph18136695. 10.3390/ijerph18136695 PMC8296866 34206264

[12] Instituto de Salud Carlos III. Análisis de los casos de COVID-19 en personal sanitario notificados a la RENAVE hasta el 10 de mayo en España. Spain: Instituto de Salud Carlos III; 2021, May 29 [cited 2023 Dec 22]. Available from: https://www.isciii.es/QueHacemos/Servicios/VigilanciaSaludPublicaRENAVE/EnfermedadesTransmisibles/Documents/INFORMES/Informes%20COVID-19/COVID-19%20en%20personal%20sanitario%2029%20de%20mayo%20de%202020.pdf.

[13] Zhang X, Jiang Y, Yu H, Jiang Y, Guan Q, Zhao W (2021). Psychological and occupational impact on healthcare workers and its associated factors during the COVID-19 outbreak in China. Int Arch Occup Environ Health.

[14] Lai J, Ma S, Wang Y, Cai Z, Hu J, Wei N (2020). Factors associated with mental health outcomes among health care workers exposed to coronavirus disease 2019. JAMA Netw Open.

[15] Lu W, Wang H, Lin Y, Li L (2020). Psychological status of medical workforce during the COVID-19 pandemic: a cross-sectional study. Psychiatry Res.

[16] Alharbi J, Jackson D, Usher K (2020). The potential for COVID-19 to contribute to compassion fatigue in critical care nurses. J Clin Nurs.

[17] Mo Y, Deng L, Zhang L, Lang Q, Liao C, Wang N (2020). Work stress among Chinese nurses to support Wuhan in fighting against COVID-19 epidemic. J Nurs Manag.

[18] Neto MLR, Almeida HG, Esmeraldo JD, Nobre CB, Pinheiro WR, de Oliveira CRT (2020). When health professionals look death in the eye: the mental health of professionals who deal daily with the 2019 coronavirus outbreak. Psychiatry Res.

[19] Creswell JW, Plano Clark V, Gutmann ML, Hanson WE, Tashakkori A, Teddlie C (2003). An expanded typology for classifying mixed methods research into designs. Handbook of mixed methods in social and behavioral research.

[20] Goldberg DP, Hillier VF (1979). A scaled version of the general health questionnaire. Psychol Med.

[21] Lobo A, Pérez-Echeverría MJ, Artal J (1986). Validity of the scaled version of the General Health Questionnaire (GHQ-28) in a Spanish population. Psychol Med.

[22] Bobes J, Calcedo-Barba A, García M, François M, Rico-Villademoros F, González MP, Bascarán MT, Bousoño M (2000). Evaluation of the psychometric properties of the Spanish version of 5 questionnaires for the evaluation of post-traumatic stress syndrome. Actas Esp Psiquiatr.

[23] Davidson JR, Book SW, Colket JT, Tupler LA, Roth S, David D, Hertzberg M, Mellman T, Beckham JC, Smith RD, Davison RM, Katz R, Feldman ME (1997). Assessment of a new self-rating scale for post-traumatic stress disorder. Psychol Med.

[24] Chen Q, Liang M, Li Y, Guo J, Fei D, Wang L, He L, Sheng C, Cai Y, Li X, Wang J, Zhang Z (2020). Mental health care for medical staff in China during the COVID-19 outbreak. Lancet Psychiatry.

[25] Flick U (1998). An Introduction to Qualitative Research.

[26] Braun V, Clarke V (2006). Using thematic analysis in psychology. Qual Res Psychol.

[27] National Statistics Institute. Profesionales Sanitarios Colegiados. In: National Statistics Institute, editor. 2021.

[28] Martínez-Caballero CM, Cárdaba-García RM, Varas-Manovel R, García-Sanz LM, Martínez-Piedra J, Fernández-Carbajo JJ, Pérez-Pérez L, Madrigal-Fernández MA, Barba-Pérez M, Olea E, Durantez-Fernández C, Herrero-Frutos MT. Analyzing the Impact of COVID-19 Trauma on Developing Post-Traumatic Stress Disorder among Emergency Medical Workers in Spain. Int J Environ Res Public Health. 2021;18(17). 10.3390/ijerph18179132. 10.3390/ijerph18179132 PMC8431006 34501726

[29] Olaya B, Pérez-Moreno M, Bueno-Notivol J, Gracia-García P, Lasheras I, Santabárbara J. Prevalence of Depression among Healthcare Workers during the COVID-19 Outbreak: A Systematic Review and Meta-Analysis. J Clin Med. 2021;10(15). 10.3390/jcm10153406. 10.3390/jcm10153406 PMC8348388 34362188

[30] Rossi R, Socci V, Pacitti F, Di Lorenzo G, Di Marco A, Siracusano A, Rossi A (2020). Mental health outcomes among frontline and second-line health care workers during the coronavirus disease 2019 (COVID-19) pandemic in Italy. JAMA Netw Open.

[31] Alonso J, Vilagut G, Alayo I, Ferrer M, Amigo F, Aragón-Peña A, Aragonès E, Campos M, Del Cura-González I, Urreta I, Espuga M, González Pinto A, Haro JM, López Fresneña N, Martínez de Salázar A, Molina JD, Ortí Lucas RM, Parellada M, Pelayo-Terán JM, Pérez Zapata A, Pijoan JI, Plana N, Puig MT, Rius C, Rodriguez-Blazquez C, Sanz F, Serra C, Kessler RC, Bruffaerts R, Vieta E, Pérez-Solá V, Mortier P. Mental impact of Covid-19 among Spanish healthcare workers. A large longitudinal survey. Epidemiol Psychiatr Sci. 2022;31:e28. 10.1017/s2045796022000130. 10.1017/S2045796022000130 PMC9069586 35485802

[32] Andhavarapu S, Yardi I, Bzhilyanskaya V, Lurie T, Bhinder M, Patel P, Pourmand A, Tran QK (2022). Post-traumatic stress in healthcare workers during the COVID-19 pandemic: a systematic review and meta-analysis. Psychiatry Res.

[33] Poon YR, Lin YP, Griffiths P, Yong KK, Seah B, Liaw SY (2022). A global overview of healthcare workers' turnover intention amid COVID-19 pandemic: a systematic review with future directions. Hum Resour Health.

[34] Liu Q, Luo D, Haase JE, Guo Q, Wang XQ, Liu S, Xia L, Liu Z, Yang J, Yang BX (2020). The experiences of health-care providers during the COVID-19 crisis in China: a qualitative study. Lancet Glob Health.

[35] Xue Y, Lopes J, Ritchie K, D'Alessandro AM, Banfield L, McCabe RE, Heber A, Lanius RA, McKinnon MC (2022). Potential circumstances associated with moral injury and moral distress in healthcare workers and public safety personnel across the globe during COVID-19: a scoping review. Front Psychiatry.

[36] Burke BL, Martens A, Faucher EH (2010). Two decades of terror management theory: a meta-analysis of mortality salience research. Pers Soc Psychol Rev.

[37] Routledge C, Juhl J (2010). When death thoughts lead to death fears: Mortality salience increases death anxiety for individuals who lack meaning in life. Cogn Emot.

[38] Ohayon MM (2002). Epidemiology of insomnia: what we know and what we still need to learn. Sleep Med Rev.

[39] Serrano-Ripoll MJ, Zamanillo-Campos R, Castro A, Fiol-de Roque MA, Ricci-Cabello I (2021). Insomnia and sleep quality in healthcare workers fighting against COVID-19: a systematic review of the literature and meta-analysis. Actas Esp Psiquiatr.

[40] Al Maqbali M, Al Sinani M, Al-Lenjawi B (2021). Prevalence of stress, depression, anxiety and sleep disturbance among nurses during the COVID-19 pandemic: a systematic review and meta-analysis. J Psychosom Res.

[41] Giurgiu DI, Jeoffrion C, Roland-Lévy C, Grasset B, Dessomme BK, Moret L, Roquelaure Y, Caubet A, Verger C, Laraqui Cel H, Lombrail P, Geraut C, Tripodi D (2016). Wellbeing and occupational risk perception among health care workers: a multicenter study in Morocco and France. J Occup Med Toxicol.

[42] Fiol-DeRoque MA, Serrano-Ripoll MJ, Jiménez R, Zamanillo-Campos R, Yáñez-Juan AM, Bennasar-Veny M, Leiva A, Gervilla E, García-Buades ME, García-Toro M, Alonso-Coello P, Pastor-Moreno G, Ruiz-Pérez I, Sitges C, García-Campayo J, Llobera-Cánaves J, Ricci-Cabello I (2021). A mobile phone-based intervention to reduce mental health problems in health care workers during the COVID-19 pandemic (PsyCovidApp): randomized controlled trial. JMIR Mhealth Uhealth.

[43] Galatea Foundation. Repercussions de la COVID sobre la salut i l'exercici de la professió dels professionals de la salut de Catalunya. 2022. Available from: https://fgalatea.org/pdf/estudis/00_resum-covid2.pdf.

[44] Lincoln YS, Guba EG. Naturalistic Inquiry. SAGE Publications; 1985.

