# Correction to: Impact of COVID-19 on mental health of health care workers in Spain: a mix-methods study

**DOI:** 10.1186/s12889-024-18312-4

**Published:** 2024-03-21

**Authors:** J. Ripoll, X. Chela-Alvarez, E. Briones‑Vozmediano, M. A. Fiol de-Roque, R. Zamanillo‑ Campos, I. Ricci‑Cabello, J. Llobera, C. Calafat‑Villalonga, M. J. Serrano‑Ripoll

**Affiliations:** 1 Primary Care Research Unit of Mallorca, Palma, 07002 Spain; 2 Grup de Recerca Atenció Primària i Promoció (GRAPP-caIB). Health Research Institute of the Balearic Islands (IdISBa), Ctra. Valldemossa, Hospital Universitari Son Espases 79, Palma, 07120 Spain; 3 Research Network On Chronicity, Primary Care and Health Promotion (RICAPPS), Palma, 07120 Spain; 4 Department of Psychology, University of the Balearic Islands, Palma, 07122 Spain; 5 Departamento de Enfermería y Fisioterapia. Grupo de estudios en sociedad, salud, educación y cultura (GESEC). Universidad de Lleida.Grup de Recerca en Cures en Salut (GRECS), Institut de Recerca Biomèdica (IRB) de Lleida, Lleida, 25001 Spain

**Correction to: BMC Public Health 24, 463 (2024).**

10.1186/s12889-024-17979-z.

The original publication of this article contained several errors, the incorrect and correct information is listed in this correction article. The publisher apologizes to the authors for the inconvenience caused by this.

## Incorrect

- X. Chela
- E. MA Briones-VozmedianoFiol-de Roque
- MJS-R and MAF-D were funded by Instituto de Investigación Sanitaria de las Islas Baleares, grant number FOLIUM17/10 (co-funded by ITS-2017 and PO FSE 2014?2020) and FOLIUM19/05 (founded by ITS-2019?003), respectively. This research did not receive any specific grant from funding agencies in the public, commercial, or not-for-profit sectors.

## Correct

- **X.** Chela-Alvarez
- E. Briones-Vozmediano2, MA Fiol-de Roque1
- MJS-R and MAF-D were funded by Instituto de Investigación Sanitaria de las Islas Baleares, grant number FOLIUM17/10 (co-funded by ITS-2017 and PO FSE 2014?2020) and FOLIUM19/05 (founded by ITS-2019?003), respectively. The Research Network On Chronicity, Primary Care and Health Promotion (RICAPPS, RD21/0001/0009) has been funded by the Carlos III Health Institute, through the NextGeneration EU funds (MRR).This research did not receive any specific grant from funding agencies in the public, commercial, or not-for-profit sectors.

