# Supplementary material for: Impact of COVID-19 on mental health of health care workers in Spain: a mix-methods study
Source: BMC Public Health. 2024 Feb 14;24:463. doi: 10.1186/s12889-024-17979-z (PMC10865523; doi:10.1186/s12889-024-17979-z)
Supplement: Supplementary file 1 — Supplementary material. [file 12889_2024_17979_MOESM1_ESM.docx]

**Additional file 1: code tree**

The code tree represents the hierarchical structure of themes derived from the data.

| **Codes** |
| --- |
| Feelings |
| Concerns |
| Sources of discomfort |
| Sources of well-being |
| Strategies to cope with discomfort |
| Outcomes |
| Relationship with the risk |
| Future behavioral changes |
| What could be done from managerial positions |
